# Risk Factors and Prevalence of Meningococcal Colonisation of Children Attending Kindergarten in Northern Hungary

**DOI:** 10.3390/microorganisms14081738

**Published:** 2026-08-07

**Authors:** Andrea Horváth, Annamária Huber, Judit Sahin-Tóth, Orsolya Dobay

**Affiliations:** Institute of Medical Microbiology, Semmelweis University, 1089 Budapest, Hungary; horvath.andrea1@semmelweis.hu (A.H.);

**Keywords:** meningococcus, asymptomatic carriage, kindergartens, vaccination

## Abstract

*Neisseria meningitidis* is a bacterium that can cause high-mortality invasive infections. Asymptomatic carriage can serve as a source of infection. This study aimed to determine the meningococcal colonisation rate and risk factors for colonisation among children aged 1–6 years attending eight childcare institutions in three regions in the Northern part of Hungary. Oropharyngeal swab specimens were obtained from 180 children, and risk factors for carriage were assessed by questionnaires filled out by parents. qPCR was applied to detect meningococci and determine the serogroup. The meningococcal colonisation rate was 15.6% (28/180). One meningococcus detected belonged to serogroup X; all others were non-groupable meningococci. Meningococcal colonisation was significantly more prevalent among girls (21.5% vs. 9.2% in boys) and children living in the Central Transdanubia region. No significant association was found with other risk factors examined. According to the parental answers, 70.0% of the children were vaccinated against meningococcus; this might be an explanation for the absence of vaccine serogroups. This is the first study to examine meningococcal carriage among young children in Hungary.

## 1. Introduction

*Neisseria meningitidis* is a bacterium causing invasive meningococcal disease (IMD) presenting as meningitis and sepsis, with a high case fatality rate (8–15%) [1]. Long-term sequelae are often experienced in survivors, including limb loss and neurologic impairment [2]. Invasive meningococcal disease is most prevalent in infants under one year old. The second most commonly affected age group is children 1–4 yo, followed by 15–24-year-olds [1].

In asymptomatic carriage, meningococci colonise the mucosal surface of the oropharynx in 10% to 35% of the general population without causing disease [3]. According to Christensen et al., carriage prevalence gradually rises during childhood, from 4.5% through about 7.7% at 10 years of age, peaking in adolescence at around 23.7%, then decreasing in adulthood [4]. Nevertheless, most carriage studies focus on adolescents and young adults, and carriage data from younger children is scarce in the literature [5]. Kindergarten-age children (i.e., 2–6 yo) are the second most commonly affected age group regarding IMD, and contrary to infants, they attend childcare institutions, where close contact with their peers allows easy respiratory transmission of *N. meningitidis*.

There are 12 capsular groups of *N. meningitidis*, six of which cause the majority of disease globally—A, B, C, W, X and Y. Infections by five of these serogroups are prevented by polysaccharide (ACYW), conjugated (ACYW) and recombinant protein vaccines (B) [6].

According to the European Centre for Disease Prevention and Control (ECDC), the notification rate for IMD in Hungary was 0.2–0.5/100 000 population in the years 2019–2023. This is similar to the EU average rate of IMD (0.1–0.5 in the same years) [1]. In Hungary, the National Centre for Public Health and Pharmacy monitors IMD prevalence and serogroups associated with invasive infections. Between 2017 and 2023, IMD was most commonly detected in infants <1 yo, followed by children 1–2 yo and then by 3–5 yo in Hungary [7]. In 2022/23, 47.5% of IMD cases (n = 10) were associated with serogroup B, 19.0% (n = 4) with C and 9.5% (n = 2) with Y, while in 23.8% of the cases, no serogroup could be identified [7].

In Hungary, meningococcal vaccination is not included in the national immunisation programme, but vaccination against serogroups B and C is recommended for all infants < 1 yo and for all adolescents and adults between 14 and 25 yo entering new communities [8]. Nevertheless, only MenC vaccination is reimbursed for children < 2 yo; hence, MenB and MenACWY vaccine coverage remains probably low.

Data on meningococcal carriage in Hungary (similarly to other European countries) are limited. Our study group has reported colonisation rates of Hungarian high school and university students for the first time [9], but no studies are available on carriage in other age groups from Hungary.

Up-to-date knowledge on prevalence and serogroup distribution of carried meningococci is important for understanding the epidemiology and transmission of *N. meningitidis* and to guide vaccination recommendations. Therefore, in this study, we aimed to identify meningococcal carriage rates, serogroup distribution and associated risk factors among children aged 1–6 yo attending childcare institutions.

## 2. Materials and Methods

### 2.1. Study Population

Children aged 1–6 years attending daycare or kindergarten and adult kindergarten teachers were included in this study. Eight childcare institutions from eight different settlements in the Northern part of Hungary participated. Sites of specimen collection differed in settlement size, ranging from capital city through mid-sizes cities to villages, representing various population types. The eight institutions were from three regions of the country: Central Transdanubia, Central Hungary and Northern Hungary (Supplementary Figure S1). Parents of the children were asked to fill in a questionnaire for risk factor analysis as part of the written consent for the inclusion of their children in the study. The questionnaire was developed specifically for this study, based on similar studies in the literature.

Overall, 220 oropharyngeal specimens were collected from 180 children and 40 kindergarten teachers between May 2022 and April 2023 (evenly distributed within this time period, excluding the summer months).

### 2.2. Sample Collection and DNA Extraction

Oropharyngeal samples were obtained from the participants with sterile swabs. Specimens were transported to the laboratory in Transwab Charcoal medium (Medical Wire & Equipment, Corsham, UK) at room temperature. The QIAamp BiOstic Bacteremia DNA Kit (Qiagen, Venlo, The Netherlands) was used for extraction of bacterial DNA from the swabs. First, the swabs were immersed in two mL of PBS for two hours, and this solution was used for further study. After centrifugation, the sediment was resuspended in 450 μL of the first solution of the kit, and the kit instructions were followed. DNA concentration was measured by a NanoDrop Lite spectrophotometer (Thermo Scientific, Waltham, MA, USA). DNA samples were stored at −80 °C until further testing.

### 2.3. Molecular Identification and Characterisation of Meningococcal Isolates

Detection of the presence of *N. meningitidis* in samples was based on detection of the species-specific *sodC* gene with RT-PCR [10]. The serogroup of *N. meningitidis* (A, B, C, X, Y, W) was determined by detection of serogroup-specific genes according to the protocol described by the WHO and CDC [11]. PCR reactions were performed in a 25 μL reaction volume in 96-well plates, in triplicate, in a qTOWER3G thermal cycler (AnalyticJena, Jena, Germany), and with a high sensitivity BioTaq polymerase (Bioline, London, UK). After initial denaturation at 50 °C for 2 min and 95 °C for 10 min, 50 cycles of 95 °C for 10 s, 60 °C for 1 min, and 40 °C for 30 s followed. Ct values ≤ 37 were considered positive. Appropriate positive and negative controls were used for all PCR reactions.

### 2.4. Statistical Analysis

Statistical analyses were performed using MedCalc for Windows, version 23.6.1. (MedCalc Software, Ostend, Belgium). Continuous variables were summarised as mean and range. Categorical variables were summarised as counts and percentages. Odds ratios (ORs) with 95% confidence intervals (CIs) were calculated to assess the association between the variables and the nasopharyngeal carriage of meningococci. The association between potential risk factors and the risk of colonisation was studied using a univariate logistic regression model. Risk factors were considered significantly associated with carriage if the *p* value was <0.05. Generative artificial intelligence has not been used in this paper.

## 3. Results

### 3.1. Baseline Characteristics

The median age of the participating children was four years, with the following distribution: <3 y (n = 37), 3–4 y (n = 59), 4–5 y (n = 53), >5 y (n = 31). They had a median of one sibling. Boys and girls were represented in almost equal numbers among the 180 participants (number of males vs. females: 87 vs. 93). Most of the children were vaccinated against at least one meningococcal serogroup (70%), most commonly against serogroup C only (33.3%) or against B and C (27.1%). The majority of the study population lived in villages (56.7%) in Central Hungary and Central Transdanubia regions. From previous infections, 11.7% of participants had otitis media, 5% had pneumonia or meningitis, and 2.8% were previously hospitalised. Recent antibiotic treatment was used in 18.3% of the children. Forty-percent of them were exposed to passive smoking at home. The baseline demographic properties of the study population are summarised in Table 1.

### 3.2. Meningococcal Carriage

Overall, the meningococcal carriage rate was 15.6% among the tested kindergarten children (28/180) (95%CI: 11.0–25.6%). None of the kindergarten teachers were carriers.

Out of the detected meningococcal strains, one belonged to serogroup X; all the other detected meningococci were non-groupable (NGM). Of note, no serogroup B strains were found.

Meningococcal colonisation was significantly more commonly detected in girls: approximately 21.5% of them carried the bacterium, whereas the carriage rate was only 9.2% among boys (odds ratio for colonisation 2.71 in girls vs. boys).

Carriage did not differ across children living in different settlement types; odds ratios for colonisation were similar among children living in the capital city, cities and small villages. However, we have found significant differences in meningococcal carriage according to regions of the country: carriage was more common in children from the Central Transdanubia region compared to Central Hungary (Table 2).

**Figure 1:**
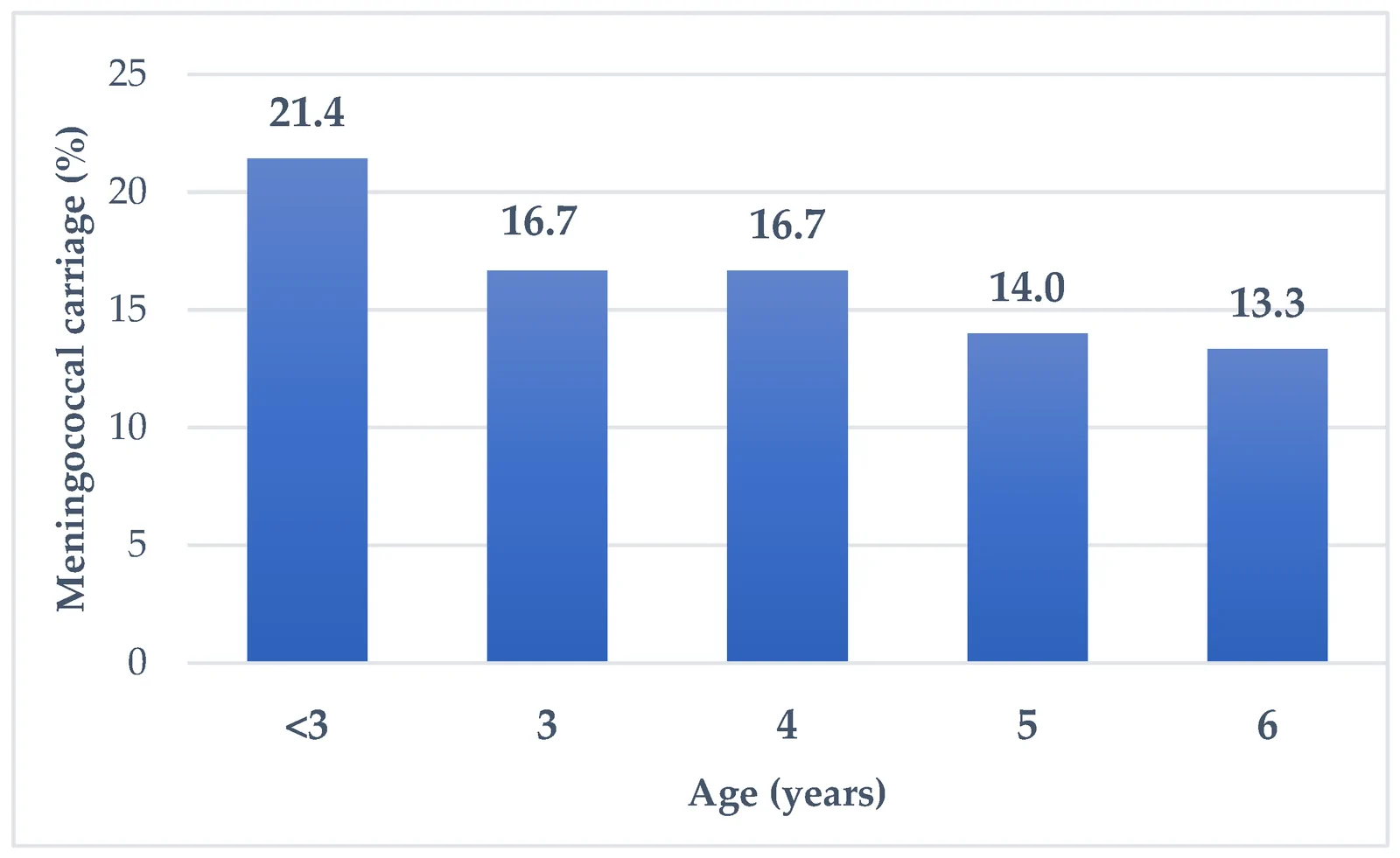
Meningococcal colonisation rate according to the age of the children.

We could detect a decreasing colonisation rate with age: under the age of 3 years, a 21.4% carriage rate was found, whereas only ≤14.0% carriage was detected in the 5–6 yo children (Figure 1). However, this was an observed, statistically non-significant trend.

The colonisation rate was higher among those who had more siblings and if the siblings were also attending childcare institutions; however, these associations were not statistically significant. Similarly, recent antibiotic treatment, having otitis media, pneumonia or meningitis, hospitalisation in anamnesis, and exposure to passive smoking had no significant effect on colonisation. Meningococcal colonisation also had no significant association with meningococcal vaccination.

## 4. Discussion

In this study, we describe the meningococcal colonisation rate and risk factors among kindergarten-aged children (1–6 yo) from Hungary. The meningococcal colonisation rate was 15.6%.

Most meningococcal carriage studies focus on adolescents and young adults, and much less data is available on carriage in younger children. Meningococcal carriage studies of pre-school children generally report colonisation rates under 10%, although these data are difficult to compare to each other due to different age groupings and different testing methodologies. In Argentina, Gentile et al. have found a 4.0% meningococcal prevalence among children 1–9 yo, lower than that of teenagers (10–17 yo, 9.4%) [12]. In Turkey, *N. meningitidis* carriage was detected in 7.0% of 0–5 yo children in 2018 and in 4.9% in 2025 [13,14]. Among children aged 5–9 yo from the Philippines, meningococcal carriage was 2.4% [15]. In Burkina Faso, 4.53% of 1–4 yo children carried *N. meningitidis* [16]. According to a meta-analysis from China, children <6 yo had a 1.01% colonisation rate [17]. In a cross-sectional study from Iceland, no meningococcal carriage was detected among children in daycare centres between 2019 and 2021 [18]. An earlier study reported a 3.2% colonisation prevalence among Dutch children in their second year of life [19]. Overall, colonisation rates were higher in our study compared to these data.

When comparing region-specific carriage prevalence in our study, meningococcal colonisation was under 10% in children from Central Hungary and Northern Hungary, in accordance with most of the literature data (9.2% and 8.3%, respectively). However, we have detected a high prevalence of meningococcal carriage in the Central Transdanubia region (23.5%). The demographic profile of children in this region differed from the overall data in our study in a few points: the vaccination rate was lower in this region (60.5% vs. 70.0%), fewer children had taken recent antibiotic therapy (11.1% vs. 18.3%) and more of them reported having pneumonia or meningitis in the past (8.6% vs. 5%). Likely, the higher-than-average meningococcal carriage prevalence in our study can be explained by a high transmission rate in the Central Transdanubia region.

In our study, the majority of carried meningococci were non-groupable; this is in accordance with findings of other studies across different age groups [12,13,18]. Vaccinations might put a selective pressure on meningococcal serogroup distribution. As there is both a monovalent (C) and a tetravalent (ACYW) vaccine against serogroup C, vaccine coverage for this type is the highest all across Europe and the US. As a result, its prevalence has decreased, and in parallel, other serogroups (W, Y, E and X) have been emerging in recent years [20]. For instance, in a large-scale study from Ireland where 16,285 young students were screened, the overall carriage rate was found to be 20.7%, and among the capsulated strains, serogroup W accounted for 10.4% [21]. In the Netherlands, a 9-fold increase in MenE was reported among young adults, four years after the implementation of the MenACWY vaccine, while the overall carriage rate of genogroupable meningococci did not change significantly [22]. Similarly, the implementation of a meningococcal A conjugate vaccine (AfriVac) across the African meningitis belt has resulted in the almost complete disappearance of MenA from vaccinated populations [23], while other serogroups have occupied the vacated niche [24].

In this study, we have detected only one groupable meningococcus, belonging to serogroup X. Generally, serogroup X is not among the most common types of meningococci; however, in some studies, it was detected at unexpectedly high rates, as in Turkey in a study from 2018 [13]. According to a meta-analysis, serogroups W and X were the most commonly carried types in the African region, and the colonisation rate was comparable in 5–10 yo to that of 11–17 yo children, the age group generally considered the most common carriers [5]. Out of the six most common causes of IMD, serogroup X is the only one not prevented by the routinely used meningococcal vaccines (ACYW and B); therefore, infections by this serogroup are expected even in vaccinated populations. Recently, the pentavalent conjugate ACWYX vaccine, the first ever vaccine to include protection against serogroup X, has been introduced in Sub-Saharan Africa [25]. In case of future widespread use of this vaccine, further serotype rearrangement is expected both among carried and invasive meningococci.

On the other hand, among carried meningococci in Europe and the US, generally serogroup B is the most common [18,21,22]. This might be explained by the fact that protein-based MenB vaccines—in contrast to the polysaccharide vaccines—have limited impact on nasopharyngeal carriage due to the antigenic variability of circulating strains [26]. As was observed by Soeters et al., even multiple doses of MenB vaccines were unable to rapidly reduce meningococcal carriage or prevent serogroup B carriage acquisition in the US [27]. A very recent American study also supports this: in the US, where the MenACWY vaccine is currently recommended for adolescents 11–12 years of age, no genogroup ACWY isolates were detected, while MenB and MenE were present in 38% and 12% of the examined population, respectively [28].

Similarly to this, the absence of the vaccine-type ACWY and the detection of a serogroup X isolate in the current study might also be associated with the relatively high vaccination rate in our study. However, the absence of serogroup B meningococci requires further explanation, especially since MenB was also found to be the most prevalent type in our previous study on high-school and university students [9]. Although the vaccination coverage in that age group was significantly lower compared to that of the children involved in the current study, as discussed above, a high vaccination rate on its own cannot automatically explain the absence of MenB. We could only speculate that (i) socioeconomic factors in our study group (high recent antibiotic use, high exposure to passive smoking) or other not explored factors might be different from participants of other studies, (ii) young children receiving multiple doses of the vaccine might gain stronger protection against MenB carriage; and (iii) maybe the meningococci circulating in Hungary share more similarity with the sequence types/clonal complexes and/or the recombinant variants of the surface antigens (factor H binding protein, Neisseria adhesin A, neisserial heparin binding antigen and porinA) of the protein-based MenB vaccine strains, and therefore the higher vaccination rate among children could more successfully also eliminate MenB from colonisation. However, we have no genotypic or antigenic data on the isolates of the current study to support this hypothesis.

Interestingly, we have found significantly higher meningococcal carriage among girls (21.5%) compared to boys (9.2%) in our study. Colonisation studies conducted among adolescents and young adults generally describe male dominance among carriers [9,21,29,30]. On the contrary, several studies report no difference in the meningococcal colonisation rate of girls and boys before adolescence [12,13,15]. However, data on carriage in this age group are limited and further studies would be required to explore if gender plays a role in meningococcal colonisation. Besides the region and female gender, other examined risk factors showed no significant association with meningococcal carriage. The bacterium was carried in higher rates among those who had more siblings and if the sibling(s) were also attending an educational institution; however, this was not a significant association. Similarly to our study, carriage was more prevalent among those who had more siblings and a higher number of household members in other reports [13,15].

A strength of the study is that this is the first report providing data on meningococcal carriage of children aged 1–6 yo in Hungary. Meningococcal colonisation data on this age group are scarce in the literature worldwide. For the interpretation of the data, it is important to consider that in this study, PCR-based detection was applied; therefore, the results could not be compared with culture-based detection studies. A limitation of the study is that geographically, the study was limited to eight institutions in three regions of Hungary; therefore, the findings are limited to the selected study sites and not representative of Northern Hungary. Furthermore, as each institution was sampled only once during the study period, spatial or temporal patterns could not be determined. Due to the limited number of carriage-positive cases, a multivariable logistic regression model was not feasible in the statistical calculations; thus, possible confounding factors were not assessed.

## 5. Conclusions

In conclusion, we have found a relatively high meningococcal carriage rate among 1–6 yo children attending childcare institutions in Hungary. The colonisation rate was particularly high among girls and those living in the Central Transdanubia region. The vast majority of the meningococci were classified as non-groupable; only one MenX and no MenB were found. Further investigations are recommended, especially to elucidate the efficacy of MenB vaccines in eliminating MenB carriage. Continuous monitoring of meningococcal carriage rates and circulating serogroups among at-risk populations is important to guide IMD prevention strategies.

## Figures and Tables

**Table 1:** Demographic characteristics of the study population.

Variables		
	**Median**	**IQR (Range)**
Age (years)	4	1 (1–6.5)
Number of siblings	1	1 (0–4)
	**Ratio (n)**	**Prevalence (%)**
Gender (Male/Female)	87/93	48.3/51.7
Otitis media (Yes/No)	21/159	11.7/88.3
Pneumonia/meningitis (Yes/No)	9/171	5/95
Recent antibiotic (Yes/No)	33/146	18.3/81.1
Hospitalisation (Yes/No)	5/175	2.8/97.2
Passive smoking (Yes/No)	72/108	40/60
Meningococcus vaccinated (Yes/No)	126/20	70/11.1
**Meningococcal vaccination serogroup**		
Only B	10	5.6
Only C	60	33.3
ACYW	2	1.1
B + C	39	21.7
ACYW + B	4	2.2
Yes, unsure of type	11	6.1
Unsure	34	18.9
No meningococcal vaccination	20	11.1
**Settlement type**		
Capital city	38	21.1
City	40	22.2
Village	102	56.7
**Region**		
Central Hungary	87	48.3
Central Transdanubia	81	45.0
Northern Hungary	12	6.7

IQR: Interquartile range.

**Table 2:** Univariate analysis of risk factors for meningococcal carriage.

	N	Meningococcus Carrier (n)	Meningococcal Carriage Rate (%)	OR	95%CI	*p*
All	180	28	15.6		11.0–21.6	
Variables						
**Gender**						
Boys	87	8	9.2	1 (ref)		
Girls	93	20	21.5	2.71	1.12–6.52	0.021 *
**Settlement type**						
Capital city	38	6	15.8	1 (ref)		
City	40	6	15.0	0.94	0.28–3.22	0.923
Village	102	16	15.7	0.99	0.36–2.76	0.99
**Region of the country**						
Central Hungary	87	8	9.2	1 (ref)		
Central Transdanubia	81	19	23.5	3.03	1.24–7.37	0.011 *
Northern Hungary	12	1	8.3	0.90	0.10–7.88	0.921
**Age**						
<3	14	3	21.4	1 (ref)		
3	30	5	16.7	0.73	0.15–3.62	0.706
4	54	9	16.7	0.73	0.17–3.17	0.683
5	50	7	14.0	0.60	0.13–2.70	0.512
6	30	4	13.3	0.56	0.11–2.95	0.503
No answer	2	0	0			
**Number of siblings**						
0	35	6	17.1	1 (ref)		
1	83	8	9.6	0.52	0.6–1.62	0.263
2	45	8	17.8	1.05	033–3.35	0.941
≥3	17	6	35.5	2.64	0.70–9.94	0.156
**Sibling(s) are attending educational institution**						
No	62	7	11.3	1 (ref)		
Yes	118	21	17.8	1.70	0.68–4.25	0.242
**Recent history of otitis media**						
No	159	22	13.8	1 (ref)		
Yes	21	6	28.6	2.49	0.87–7.10	0.104
**History of pneumonia/meningitis**						
No	171	27	15.8	1 (ref)		
Yes	9	1	11.1	0.59	0.07–4.84	0.599
**Recent antibiotic treatment**						
No	146	22	15.1	1 (ref)		
Yes	33	6	18.2	1.26	0.47–3.41	0.651
No answer	1	0	0			
**History of** **hospitalisation**						
No	175	17	15.4	1 (ref)		
Yes	5	1	20.0	1.37	0.15–12.74	0.788
**Exposure to passive smoking**						
No	108	15	13.9	1 (ref)		
Yes	72	13	18.1	1.34	0.61–3.07	0.453
**Meningococcal vaccination**						
B	10	2	20.0			
C	60	10	16.7			
B + C	39	11	28.2			
ACWY	2	0	0			
ACYW + B	4	0	0			
Yes, unsure of type	11	1	9.1			
Unsure	34	2	5.9			
No	20	2	10.0	1 (ref)		
Yes, any type	126	24	19.0	2.12	0.46–9.75	0.297

*: Significant association.

## Data Availability

The original contributions presented in this study are included in the article/Supplementary Material. Further inquiries can be directed to the corresponding author.

## Supplementary Materials

The following supporting information can be downloaded at: https://www.mdpi.com/article/10.3390/microorganisms14081738/s1, Figure S1: Location of the sample collection on the map of Hungary.

## Abbreviations

The following abbreviations are used in this manuscript:

qPCR	quantitative PCR
IMD	invasive meningococcal disease
ECDC	European Centre for Disease Prevention and Control
EU	European Union
CDC	Centers for Disease Control and Prevention
WHO	World Health Organization
MenA/B/C, etc.	meningococcal serogroup A/B/C, etc.
NGM	non-groupable meningococci
yo	years old
OR	odds ratio
CI	confidence interval
