# Supplementary material for: Risk Factors and Prevalence of Meningococcal Colonisation of Children Attending Kindergarten in Northern Hungary
Source: Microorganisms. 2026 Aug 7;14(8):1738. doi: 10.3390/microorganisms14081738 (PMC13515711; doi:10.3390/microorganisms14081738)
Supplement: Supplementary file 1 [file microorganisms-14-01738-s001.zip › microorganisms-4440123-supplementary.pdf]

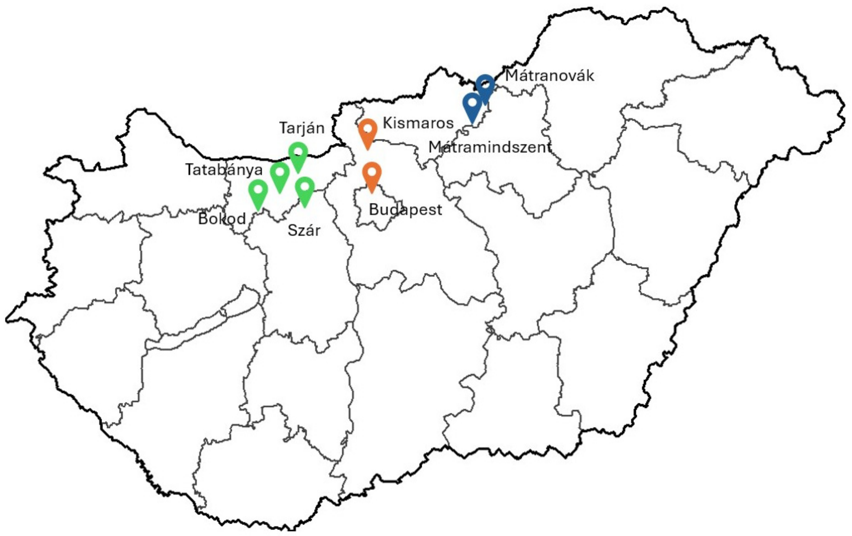

Supplementary Figure S1. Location of the sample collection on the map of Hungary

Green, Central Transdanubia; orange, Central Hungary and blue, Northern Hungary.

Modified from https://hu.wikipedia.org/wiki/F%C3%A1jl:Hungary_map_with_Balaton.png, under Creative Commons 3.0 Unported licence
